# Public health nurse advocacy as essential for addressing the health impacts of climate change

**DOI:** 10.3389/phrs.2026.1609391

**Published:** 2026-09-21

**Authors:** Salimah R. Walani, Shahin Kassam, Zahra Shajani, Aliyah Dosani

**Affiliations:** 1 School of Nursing and Midwifery, Aga Khan University, Karachi, Pakistan; 2 Faculty of Health and Social Development, School of Nursing, The University of British Columbia Okanagan, Kelowna, BC, Canada; 3 University of Calgary, Calgary, AB, Canada; 4 School of Nursing and Midwifery, Faculty of Health, Community and Education, Mount Royal University, Calgary, AB, Canada; 5 Department of Community Health Sciences, Cumming School of Medicine, University of Calgary, Calgary, AB, Canada; 6 O’Brien Institute for Public Health, University of Calgary, Calgary, AB, Canada

**Keywords:** advocacy, call to action, climate action, policy analysis, public health nursing

## Abstract

**Background:**

Climate change is transforming the environmental, social and structural determinants of health, intensifying inequities across populations and geographies. Although nurses are at the forefront of addressing climate-related health impacts, nurses’ influence on climate policy remains significantly underleveraged. Strengthening the leadership and advocacy of nurses is essential for meeting the escalating population health challenges posed by climate change.

**Analysis:**

There is limited organized advocacy for climate action by public health nurses. At the 7th Global Network of Public Health Nursing (GNPHN) Conference, we invited nurses from diverse backgrounds to share their perspectives on climate-related research, practice, and policy.

**Policy Options:**

The participants agreed to form a global coalition to enhance climate advocacy by public health nurses. Here, we outline four key calls to action emerging from conference discussion: (1) building inclusive research through transdisciplinary collaboration, (2) dismantling inequities by integrating gender and race into climate governance, (3) promoting language consistency through intersectional and equity-oriented frameworks, and (4) integrating climate change content into nursing education.

**Conclusion:**

GNPHN conference mobilised public health nurses, creating a platform and transforming awareness into collective action by outlining four calls to action for advancing climate action and advocacy by nurses.

## Background

Climate change has become a global public health threat [1]. Recent evidence shows that rising temperatures, extreme weather events, air pollution, environmental degradation, and the spread of infectious diseases have caused adverse health outcomes and have exacerbated inequities among populations [1]. The importance of addressing the negative impact of climate change and the need to improve resilience of populations against intensifying threats was recognized as early as in 1992 with the United Nations (UN) Framework Convention on Climate Change [2]. However, the urgency to combat the impact of climate change gained momentum with the UN Sustainable Development Goals 2015–2030 [3]. Sustainable Development Goal # 13 calls upon the member nations to increase investments to address the impact of climate change and to increase capacity and education to help mitigate the effects of climate-related hazards [3]. Collectively, these developments highlight the critical necessity for coordinated, multisectoral action to safeguard population health against climate instability.

The link between climate change and population health has been well-documented. The World Health Organization (WHO) highlights that climate hazards are undeniably associated with adverse maternal and newborn health outcomes, such as gestational diabetes, hypertensive disorders of pregnancy, preterm birth, low birth weight, and stillbirth [4]. Additionally, exposure to climate hazards affects mental health, contributing to stress, anxiety, and depression [4]. Another irrefutable result of climate change is exacerbation of inequalities in healthcare access and health outcomes of people from high-income and low-income countries. Heat waves, floods and storms are directly and indirectly increasing not only mortality and morbidity, but health inequities within and between nations. Consequently, climate change is hampering and reversing the global efforts to reduce inequities among populations [5]. While people in lower income countries suffer increased vulnerability to the adverse effects of climate change, most climate-related research and advocacy efforts have been conducted in high-income countries [6].

Public health professionals, particularly nurses must engage in collective and organized actions to confront the health challenges posed by climate change. Together, we must deliberately advocate for environmental justice within the global climate agenda and prepare ourselves and the populations to be climate resilient. The objective of this paper is to propose policy actions from the platform of the Global Network of Public Health Nursing (GNPHN) to enable public health nurses to engage actively with the climate agenda.

### The need for a global platform for public health nurses to engage in climate advocacy

In 2008, the International Council for Nurses (ICN) released its first position statement, addressing nursing practice and climate change. This statement was revised in 2018 and then recently in 2024, to align the ICN’s position with the growing knowledge of climate-related inequities and the evolving role of nurses [7]. The 2024 ICN statement declared climate change as the ‘single greatest health threat’ and called upon nurses to engage in global climate policy dialogue to advocate for evidence-informed action-oriented strategies.

Despite the ICN’s statement, evidence suggests that collective nurse-led advocacy groups remain limited and are primarily located in high-income countries. There are only a few collective advocacy groups led by nurses and they are mainly located in high-income countries. The Canadian Association of Nurses and the Environment (CANE), and the Alliance of Nurses for Healthy Environments (ANHE) are two such groups, demonstrating collective advocacy by nurses.

As a non-profit association, CANE commits to promoting planetary health and foster action-oriented advocacy for health and wellbeing of human and the environment [8]. CANE advocates for revision of the Canadian Environmental Protection Act (CEPA) and “Right to a Healthy Environment” while building capacity of nursing to influence policy change [8]. Similarly, ANHE envisions engagement with nurses globally to advance environmental justice [9]. As a professional organization, ANHE employs policy advocacy as a central, multi-pronged platform. Monthly transdisciplinary webinars provide policy-oriented resources and training to nurses. Partnering with environmental health coalitions and providing input on policy guidelines are among some of the advocacy approaches used by ANHE [9]. Both these organizations are based in North America and are limited in geographical context with limited or no engagement with nurses from low-and middle-income countries–countries that are the most negatively impacted by climate change.

A recent article published in 2024 [10] by Gaudreau et al. Substantiates the need for nurses to engage in addressing health inequalities and emphasized that nurses can play an essential role in climate advocacy.

Evidence indicates that although many nurses support climate health action, their collective engagement in influencing climate-informed policy, practice, and systems remains limited [10, 11].

### An opportunity to advance policy action by public health nurses

Public health nurses are well-positioned to address climate change by highlighting the environmental injustice and by mobilizing resources to improve access to timely care, response and social services needed by those affected by climate-related adverse outcomes. While urgent calls by nursing colleagues [11–13] for increasing advocacy and involvement of nurses in the climate agenda have been made, organised policy advocacy is often overlooked by nurses [14, 15]. Recognizing the power and potential of nurses to respond to climate and planetary health crisis, the authors of this paper organized a session on the role of public health nurses in responding to climate and environmental change at the 7th GNPHN Conference held in July 2025, in Calgary, Canada. In this policy brief, we present a set of calls to action, discussed by the attendees of the session.

GNPHN is a membership-based organization that brings together public health nurses and community health nurses to work together on strengthening practice, policy, research, leadership and education to increase access to public health services globally [16]. GNPHN conferences began in 2009 to discuss global health issues from the perspective of public health nursing practice [16]. The 7th GNPHN conference was attended by over 200 nurses from 15 countries, and it served as a platform to engage public health nurses from around the world, to share their work and knowledge on various global health topics, including the impact of climate change on health.

Using the GNPHN as an opportunity, we (SW, SK) conducted a pre-planned 90-min, interactive session, titled “THINK TANK: Role of nurses in mitigating the impact of climate change on population health outcomes”. The session was attended by 28 participants who held various roles in health practice, education, leadership, research, and policymaking. They represented a diverse global nursing community and came from Canada, Japan, Norway, Liberia, Ghana, the United Kingdom, Pakistan, Republic of Korea, England, Kenya, the USA, Ireland, Nigeria, Australia, Barbados, Ethiopia, Haiti, Rwanda, South Africa, and Sweden. This wide geographic representation united these nurses in their global commitment to addressing climate-related health risks.

In the first 20 min of the session, SW and SK presented a set of slides that included the details of global efforts to address climate change, the current nurse-led organizations in the climate space and some published literature urging nurses to engage in climate action.

The participants discussed International Council of Nurses (ICN) Position Statement (2024), titled “Nurses, climate change and health” [8] while collectively and critically examined the role of PHNs in climate advocacy. During the session, the participants shared their personal experiences and perspectives related to the role of public health nurses in climate action and they agreed to establishing an international working group within the GNPHN to coordinate training and resource-sharing, and a commitment to advocate jointly for stronger policy frameworks supporting nurses in climate-affected communities. The participants also highlighted the importance of mental health support for nurses working under extreme conditions and agreed to creating an accessible digital platform for peer support and knowledge exchange.

### Building consensus for global engagement in climate change policy action

During the GNPHN conference, it became evident that although nurses are engaged in conducting research and participating in responding to disasters and displacement caused by climate change, there is a gap in organized advocacy by public health nurses for promoting the climate agenda, particularly in low-and middle-income countries (LMICs). Participants expressed that LMICs are home to Indigenous Peoples whose traditional knowledge, lived experiences, and leadership are essential for developing culturally responsive and locally relevant climate and health policies. Meaningful engagement of these communities should therefore be considered an integral component of equitable climate governance.

The participants agreed that there was a need to accelerate the leadership of public health nurses in addressing climate action and policy initiatives. The participants then discussed a set of four distinct calls to action, described below, for advancing climate action by public health nurses through a collaborative effort including nurses from LMICs.

## Policy options

### Building inclusive research through transdisciplinary collaboration

There is an urgent need for robust inquiry focused on the impact of climate change on health, particularly among populations experiencing inequitable vulnerability [17]. Climate research should be socially inclusive with climate change-focused datasets that allow disaggregation for race, gender, socioeconomic status, geography and additional social constructs [18]. We call on researchers, academic institutions, and policymakers to generate quantitative and qualitative data that provide depth in terms of epidemiology as well as meaningful, community-based insights that are necessary for effective context-specific interventions that can effectively respond to climate challenges for people in LMICs who are most vulnerable and impacted by climate change. We further call upon early career nurse researchers and innovators to engage actively in researching the impact and mitigation strategies.

Representation, experiences and knowledge of Indigenous populations from low- and middle-income countries must be included in climate research. Inclusion of Indigenous methods holds significant power in informing climate policymaking [19]. We call on institutions to mandate such inclusion and engage in meaningful knowledge sharing.

We call on health systems to support integration of public health nurse perspectives within climate-change focused research. Scientific evidence on public health nurses’ perceptions and strategies for developing climate-resilient health systems globally [11]. Shaping policy with public health nursing insights will improve prioritization of populations traditionally marginalized by systems on the basis of race, gender, class, social location, age, and ability [20, 21].

Public health nurses, particularly in LMICs require institutional support to convene and collaborate across health and other disciplines engaged in climate justice and planetary health equity-oriented research. We call on health and academic institutions to engage with public health nurse’s expert knowledge through their direct involvement in climate research.

### Dismantling inequity by integrating gender and race in climate governance

Environmental racism continues to drive stark health inequities, with low-income communities and under-resourced populations bearing the brunt of pollution, climate disasters, and toxic exposures [22]. Public health nurses, through their trusted roles, community presence, and commitment to health equity, are uniquely positioned to confront these injustices [20, 22]. We call on health systems, educational institutions, and policymakers to formally recognize and support the inclusion of voices and languages of diverse groups in climate decision-making and crafting of policies to ensure that the response is environmentally effective and racially and socially just.

Climate and health policy must integrate anti-oppressive frameworks to address systemic racism, colonialism, sexism, and additional forms of inequity that shape planetary health outcomes. We call on the collective involvement of public health nurses from all countries in climate policy design and evaluation processes using intersectionality-framed analytical methods [23].

### Advancing language consistency through equity-oriented frameworks and lenses

Public health nurses must develop a clear understanding of the difference between climate adaptation and climate mitigation within policy to effectively advocate for and implement health strategies that both protect communities from current climate impacts and prevent future harm. We call on nursing leaders to guide local and global nursing practice to engage in climate discourse using consistent, clear and more accessible language. By grounding climate discourse within diverse care environments, nurses must make informed, evidence-based actions that build climate resilience and promote environmental justice [24].

Public health nurses must unite in a coordinated effort to define and integrate planetary health principles within nursing practice, education, and policy [22, 25]. Public health education of these principles and translation of knowledge into practice should be deliberate and must be brought into public domain through social media and traditional broadcasting mechanisms.

### Integrating climate change agenda into nursing curricula and education

In response to the growing threat of climate change on population health, nursing education programs must require nursing students to demonstrate knowledge of climate-related impacts on human health [20, 22]. We recommend including concepts on policies that influence and shape climate health equity to help nurses develop an understanding of the links between climate change and risks of heat-related illness, respiratory and cardiovascular conditions, infectious disease, mental health challenges, food and water insecurity, and displacement. Integrating these competencies into nursing curricula worldwide is essential to prepare a workforce capable of addressing climate-driven health disparities and advocating for policies that advance climate health equity.

The integration of climate change into nursing education has been unacceptably slow, despite its profound and growing impact on human health [20]. To prepare nurses for a climate-altered future, curricula must embed climate science, environmental and planetary health principles, and the interconnectedness of people, the planet, and the shared environment, including how these relationships are shaped by policy [11, 25]. Storytelling offers a culturally responsive pedagogy that bridges scientific knowledge and lived experience, fosters empathy, and amplifies the voices of frontline communities disproportionately affected by climate change. By leveraging storytelling alongside curriculum that promotes critical reflection on policy and environmental systems, nursing education can cultivate climate-literate, equity-focused nurses. Nursing education should equip nurses to analyze the impact of policies on health systems, across clinical, community, and policy contexts—ultimately advancing population health, planetary health, and overall wellbeing [26, 27].

We call upon academic nursing leaders to create nursing specializations, certifications and degree programs that allow deeper studies of the impact of climate on health. Graduate students should be encouraged and mentored to ask research questions and conduct quantitative and qualitative studies related to climate for their theses and dissertations. Extensive mentoring for climate action should be built into the program of studies. This action is even more important for education programs that are preparing nurses to work in low-and middle-income countries, with Indigenous communities and in global public health fields.

## Conclusion

Global health networks and conferences are critical for knowledge exchange, collaboration, and building advocacy, yet their full potential in addressing climate and health challenges is only beginning to be realized. The 7th GNPHN Conference in Calgary, Canada united public health nurses from 28 countries, demonstrating the power of cross-border collaboration to accelerate climate-focused nursing education, advocacy and policy influence to create climate resilient health systems.

In this paper we urge public health nurses to lead climate advocacy and action. As Gaudreau et al., concluded in their review [10] of the recommendations from nursing associations, the current recommendations are inadequate in addressing health inequities and in promoting action by nurses’ associations. Through the platform of the 7th GNPHN conference, we call on governments, health institutions, nursing organizations, and international bodies to partner with GNPHN and public health nurses to prioritize advocacy for promoting equity and inclusion in the climate agenda. Together, we can protect population health, advance equity, and ensure that no community is left behind in the face of climate change.

## Data Availability

The original contributions presented in the study are included in the article/supplementary material, further inquiries can be directed to the corresponding author.
